# Splenic volume as a predictor of treatment response in patients with non-small cell lung cancer receiving immunotherapy

**DOI:** 10.1371/journal.pone.0270950

**Published:** 2022-07-07

**Authors:** Francesca Castagnoli, Simon Doran, Jason Lunn, Anna Minchom, Mary O’Brien, Sanjay Popat, Christina Messiou, Dow-Mu Koh

**Affiliations:** 1 Department of Radiology, Royal Marsden Hospital, Sutton, United Kingdom; 2 Division of Radiotherapy and Imaging, Institute of Cancer Research, London, United Kingdom; 3 Drug Development Unit, Royal Marsden/Institute of Cancer Research, Sutton, United Kingdom; 4 Lung Unit, Royal Marsden Hospital/Institute of Cancer Research, Sutton, United Kingdom; 5 Lung Unit, Royal Marsden Hospital/Institute of Cancer Research, London, United Kingdom; Goethe University Hospital Frankfurt, GERMANY

## Abstract

**Introduction:**

The spleen is a lymphoid organ and we hypothesize that clinical benefit to immunotherapy may present with an increase in splenic volume during treatment. The purpose of this study was to investigate whether changes in splenic volume could be observed in those showing clinical benefit versus those not showing clinical benefit to pembrolizumab treatment in non-small cell lung cancer (NSCLC) patients.

**Materials and methods:**

In this study, 70 patients with locally advanced or metastatic NSCLC treated with pembrolizumab; and who underwent baseline CT scan within 2 weeks before treatment and follow-up CT within 3 months after commencing immunotherapy were retrospectively evaluated. The splenic volume on each CT was segmented manually by outlining the splenic contour on every image and the total volume summated. We compared the splenic volume in those achieving a clinical benefit and those not achieving clinical benefit, using non-parametric Wilcoxon signed-rank test. Clinical benefit was defined as stable disease or partial response lasting for greater than 24 weeks. A p-value of <0.05 was considered statistically significant.

**Results:**

There were 23 responders and 47 non-responders based on iRECIST criteria and 35 patients with clinical benefit and 35 without clinical benefit. There was no significant difference in the median pre-treatment volume (175 vs 187 cm^3^, p = 0.34), post-treatment volume (168 vs 167 cm^3^, p = 0.39) or change in splenic volume (-0.002 vs 0.0002 cm^3^, p = 0.97) between the two groups. No significant differences were also found between the splenic volume of patients with partial response, stable disease or progressive disease (p>0.017). Moreover, there was no statistically significant difference between progression-free survival and time to disease progression when the splenic volume was categorized as smaller or larger than the median pre-treatment or post-treatment volume (p>0.05).

**Conclusion:**

No significant differences were observed in the splenic volume of those showing clinical benefit versus those without clinical benefit to pembrolizumab treatment in NSCLC patients. CT splenic volume cannot be used as a potentially simple biomarker of response to immunotherapy.

## Introduction

Immunotherapy is one of the most important breakthroughs in cancer treatment, and, compared with standard therapies, immune checkpoint inhibitors targeting programmed cell death 1 (PD-1) have been found to significantly prolong overall survival (OS) in patients with many different tumor types [1, 2]. Since the Food and Drug Administration (FDA) first approved PD-1 inhibitors in 2016 for the treatment of non-small cell lung cancer (NSCLC), pembrolizumab-based immunotherapy has been become standard of care [3–5].

Conventional chemotherapy has significant adverse effects on hematopoiesis and immunocompetence, altering the splenic niche and spleen volume [6–8]. For example, it was demonstrated that low dose administration of several chemotherapeutic agents such as gemcitabine and 5-fluorouracil can prevent the accumulation of myeloid derived suppressor cells (MDSCs) by restoring CD8+ T cells in the spleen [7]. Other studies recently proposed that the spleen can be used as a barometer of systemic immune response during immunotherapy as they observed that anti- programmed death-ligand 1 (PD-L1) treatment increased the percent population of monocytes/macrophages, CD8+ cells and natural killer cells in the spleen [9, 10]. It was also shown that highly dosed ipilimumab plus nivolumab resulted in increased proliferation of CD4+ and CD8+ lymphocytes as well as an increase of activated T cells and central memory T lymphocytes in the spleen which was accompanied by increased size of this organ [11].

Moreover, establishing better predictive markers for immunotherapy in lung cancer is a large unmet clinical need with tumoral PD-L1 (TPS) being an imperfect biomarker. In NSCLC patients with TPS of >50% the response rate to pembrolizumab is 44.8% and in NSCLC patients with PD-L1 <50% the response to pembrolizumab-chemotherapy combination is 56.7% [12, 13]. Therefore, using the current method of patient selection, around 50% of patients are expected to not respond to immunotherapy.

We hypothesize therefore, in patients without gross tumor infiltration of the spleen, changes in splenic volume during treatment may be correlated with alterations in the number and function of immune cells in the spleen. Although studies have investigated the correlation between the use of chemotherapy and the spleen volume [14, 15] and between immunotherapy and other types of tumour [16], to our knowledge, no reports have described the changing patterns of splenic size during immunotherapy in lung cancer patients, or their clinical significance. Therefore, the aim of our study was to investigate whether changes in splenic volume could be observed in patients that showed clinical benefit versus those who did not show clinical benefit to pembrolizumab treatment in patients with NSCLC.

## Materials and methods

### Study population

This retrospective study was approved by the institutional review board of the Royal Marsden Hospital. The requirement for written informed consent was waived. The inclusion criteria were as follows: (1) histologically proven NSCLC; (2) primary tumor with a minimum diameter >2cm; (3) no prior thoracic radiotherapy or surgery for the lung tumour; (4) underwent a baseline CT within 2 weeks before treatment and follow-up CT within 3 months after commencing immunotherapy.

We analyzed and divided the patients according to clinical benefit and iRECIST criteria [17]. Clinical benefit (CB) was defined as stable disease or partial response lasting for greater than 24 weeks, no clinical benefit as stable disease or partial response lasting less that 24 weeks or disease progression. Responders were defined as those showing a complete or partial response by iRECIST, while non-responders were defined as those showing stable or progressive disease at 12 weeks after therapy. For each patient, we also recorded their time to progression after initiating treatment, as well as time of death (if it occurred).

### Computed tomography

Portal venous (PV) phase CE-CT were performed in all patients. All the CE-CT were performed at our institution using Siemens CT scanners (SOMATOM Definition Flash and SOMATOM Definition Edge; Siemens Healthcare, Erlangen, Germany). The thorax-abdomen CT were performed with iodine-based intravenous contrast (3–5 ml/second, total amount of 90–150ml, followed by bolus injection of 30cc normal saline) in portal-venous phase at 70 seconds delay. Images from all CT scanners were reconstructed at 3 mm slice thickness, which were used for splenic segmentation.

### Measurement of splenic volume

**Fig 1:**
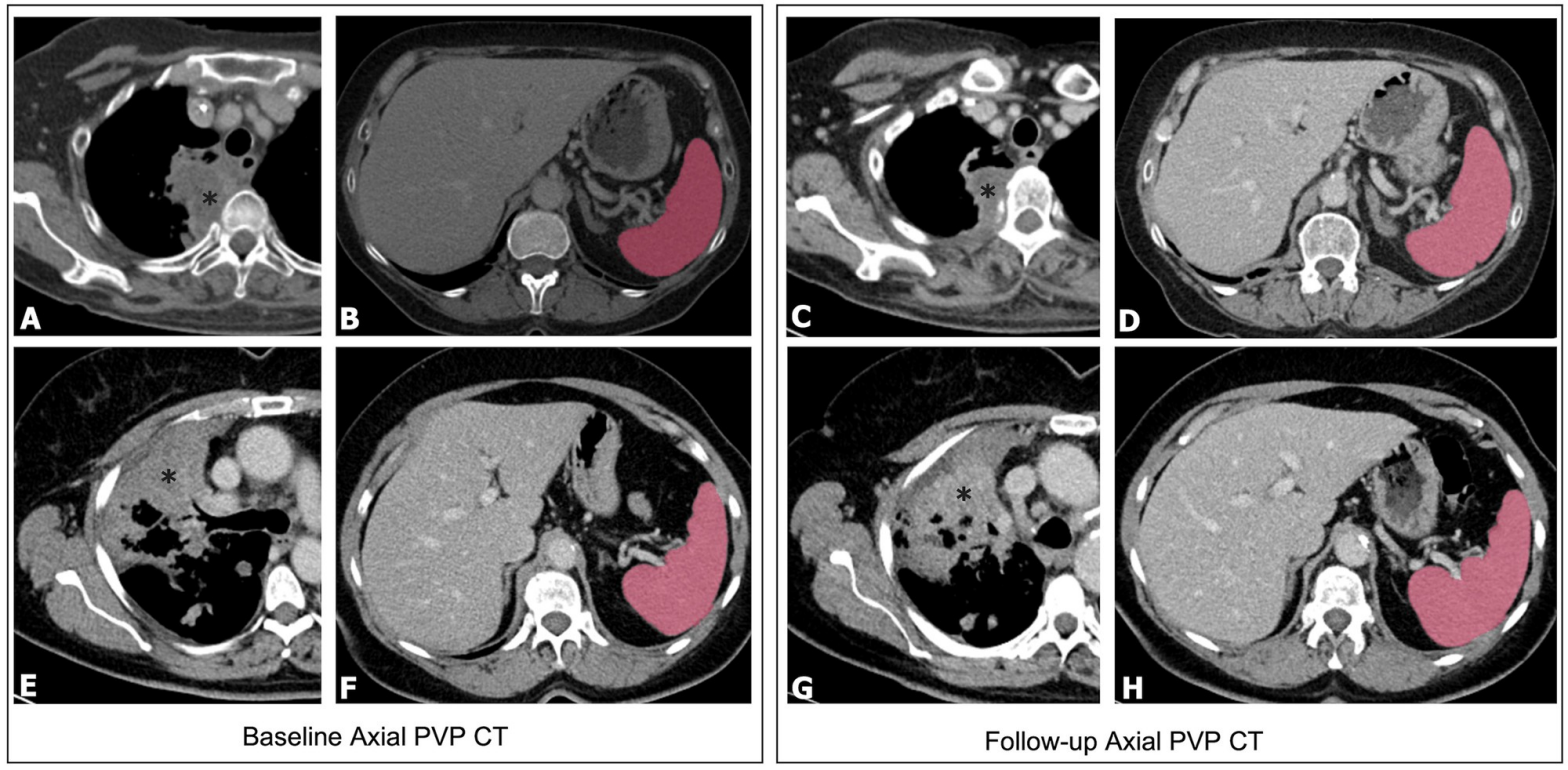
Representative computed tomography (CT) images used to calculate spleen volume. Non-small cell lung cancer (*). Baseline axial CT scans and follow-up scans in a responder patient (A-D) and a non-responder (E-H).

The splenic volume was segmented manually on each CT by a radiologist (F.C. with 5 years of experience in abdominal imaging) using OHIF DICOM viewer on our XNAT research PACS system, by outlining the splenic contour on every relevant image and the total volume was summated using an offline Matlab script (Fig 1).

### Statistical analysis

We compared the splenic volume in patients with and without clinical benefit; and between responders and non-responders using non-parametric Wilcoxon signed-rank test. Time to disease progression were calculated as the time from the start of treatment to disease recurrence/progression or death. Time to progression were compared in patients with clinical benefit to treatment versus those without benefit to treatment, as well as responders versus non-responders, stratified using the median pre-treatment splenic volume, post-treatment splenic volume and change in splenic volume using the Kaplan–Meier method. All statistical analyses were performed with MedCalc (version 12.1.0 for Microsoft Windows 2000/XP/Vista/7; MedCalc Software, Mariakerke, Belgium). A p-value less than 0.05 was considered statistically significant.

## Results

Initially, 105 patients were identified; 29 patients were excluded due to lack of baseline imaging (n = 14) or follow-up scans (n = 15); 2 patients were excluded for suboptimal scan quality and 4 other patients were excluded due to lack of clinical follow-up. The remaining 70 patients constituted our final study population. The included patients had irresectable/advanced stage NSCLC and received pembrolizumab between March 2019 and May 2020.

32 males and 38 females (median age = 68.71, range = 51–92 years) were enrolled. The majority (n = 69, 98.6%) had American Joint Committee on Cancer (AJCC) stage III or stage IV disease. The demographic and clinical characteristics of patients in this study are shown in Table 1.

**Table 1:** Patient demographic and tumor characteristics.

Characteristics (N = 70)	Number	Percentage, %
Age (median)	68.7	
Range	51–92	
Sex (female/male)	38/32	
Histology		
Adenocarcinoma	56	80
Squamous cell carcinoma	10	14.3
Mixed adenocarcinoma/squamous cell carcinoma	1	4.3
Carcinoma—other	3	1.4
T stage		
T1	0	0
T2	1	1.4
T3	13	18.6
T4	56	80
Regimen administered		
Pembrolizumab	59	84.3
Pembrolizumab, carboplatin, pemetrexed	11	15.7

There were 1 complete response, 22 partial response, 23 stable disease and 24 progressive disease. There were 23 responders and 47 non-responders. Patients with stable disease or partial response lasting for greater than 24 weeks were deemed to have clinical benefit (n = 35) and the rest as without clinical benefit (n = 35).

### Splenic volume analyses before and after treatment

Overall, the median splenic volume at baseline and at week 12 of pembrolizumab did not show any significant volume changes. After 3 months, the median spleen volume was 204 cm^3^ (range: 42.5–455 cm^3^) when compared with the baseline volume (201 cm^3^ [39.6–467 cm^3^ range]).

**Fig 2:**
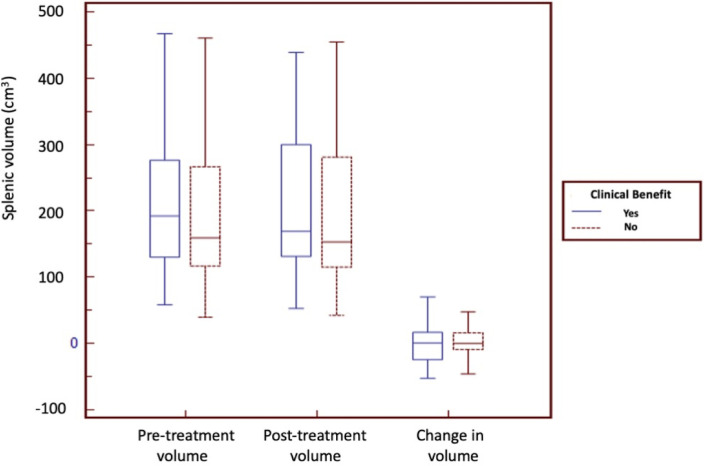
Splenic volume does not show significant changes in NSCLC patients with and without clinical benefit treated with pembrolizumab. The boxplots show the median spleen volume of NSCLC treated with pembrolizumab at baseline, after 12 weeks of treatment and the change in splenic volume between patients with and without clinical benefit.

**Fig 3:**
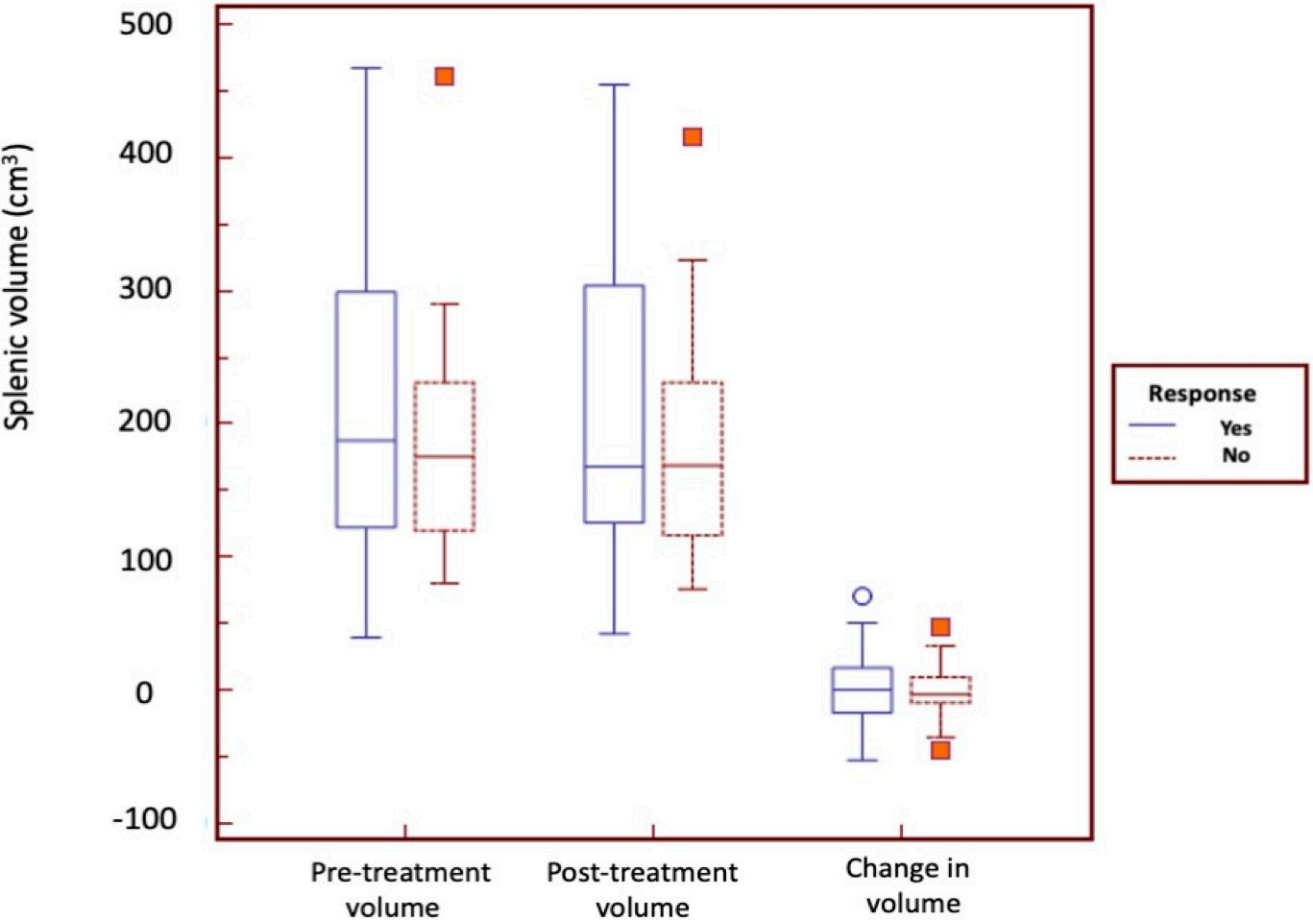
Splenic volume does not show significant changes in responders and non-responders. The boxplots show the median pre-treatment splenic volume, post-treatment splenic volume or change in splenic volume between responders and non-responders.

We found no significant difference in the median pre-treatment splenic volume (159 vs 192 cm^3^, p = 0.36), post-treatment splenic volume (153 vs 169 cm^3^, p = 0.35) or change in splenic volume (0 vs 0.0008 cm^3^, p = 0.97) between patients with clinical benefit and those without clinical benefit (Fig 2). We also found no significant difference in the median pre-treatment splenic volume (175 vs 187 cm^3^, p = 0.34), post-treatment splenic volume (168 vs 167 cm^3^, p = 0.39) or change in splenic volume (-0.002 vs 0.0002 cm^3^, p = 0.97) between responders and non-responders (Fig 3).

**Fig 4:**
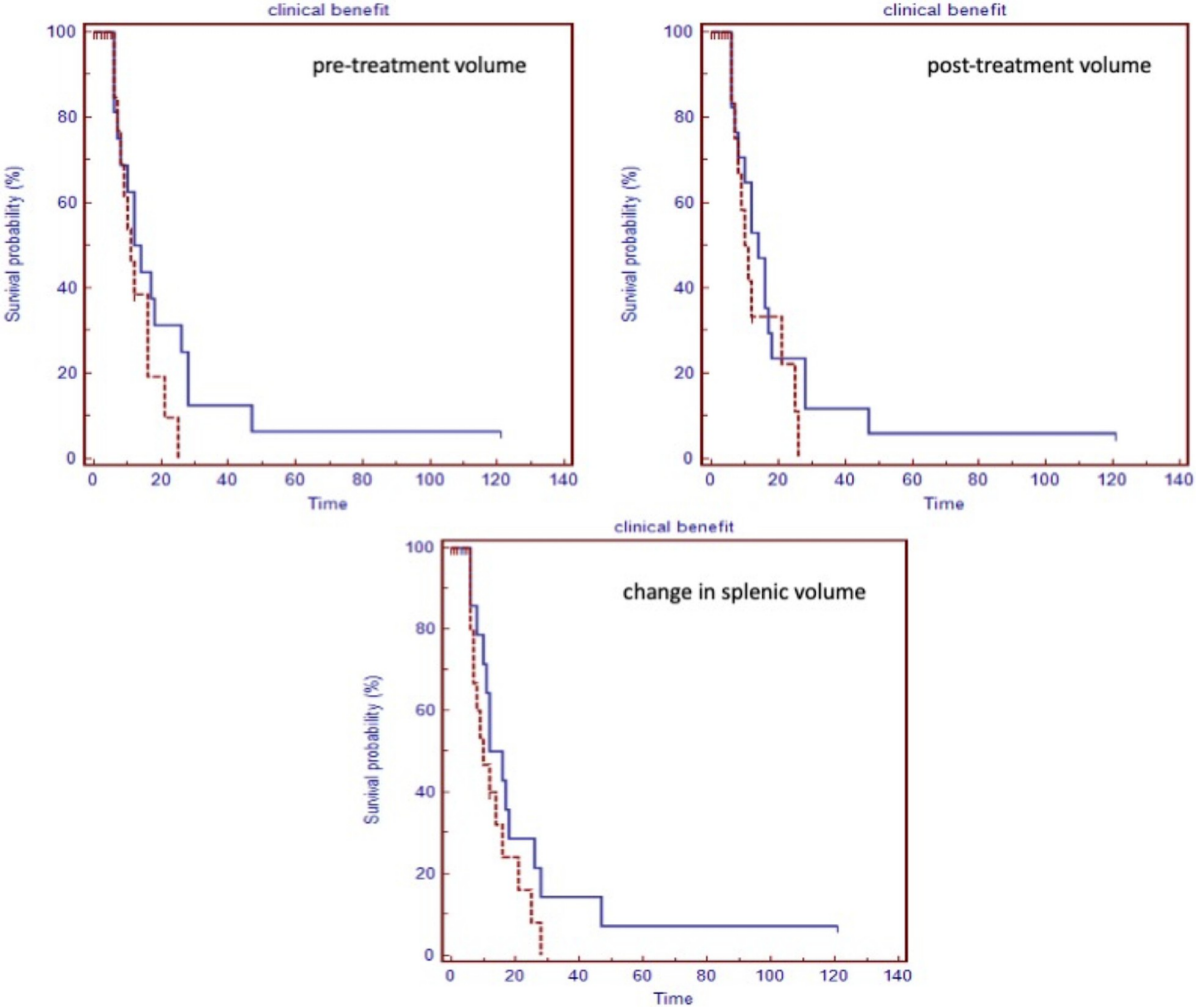
Kaplan-Meier curves for time to disease progression. The Kaplan-Meier curves show the median time to progression between patients with clinical benefit to treatment versus those without benefit to treatment stratified using the median pre-treatment splenic volume, post-treatment splenic volume and change in splenic volume.

Moreover, there was no statistically significant difference in the median time to progression between patients with clinical benefit versus those without clinical benefit stratified by the median pre-treatment volume (12 months versus 11 months, p = 0.16), median post-treatment volume (14 months versus 10 months, p = 0.29) and median change in splenic volume (12 months versus 10 months, p = 0.18) (Fig 4).

There was also no statistically significant difference in the median time to progression between responders and non-responders stratified by the median pre-treatment volume (18 months versus 16 months, p = 0.68), median post-treatment volume (16 months versus 25 months, p = 0.72) and median change in splenic volume (17 months versus 16 months, p = 0.78).

## Discussion

Pembrolizumab is a PD-1 receptor inhibitors whose efficacy has been demonstrated in many clinical trials [18–20]. It has been approved for the treatment of NSCLC and melanoma by the FDA.

Monitoring the impact of immunotherapy on the spleen, an organ known to regulate hematopoiesis and immune responses [21], is of interest in the context of appreciating the effects immunotherapy in NSCLC and other epithelial malignancies.

To our knowledge, this is first reported study that analyzed splenic size changes measured by 3-D volumetry as a potential indicator of alterations of the immune function during immunotherapy and its potential clinical significance. However, our study did not show any significant change in splenic volumetric during treatment with pembrolizumab, between the baseline measurement and follow-up measurement on CT, and hence there was no observable correlation with treatment outcomes.

Wen et al. [15] reported early decrease in splenic volume after the commencement of platinum-based chemoradiotherapy in patients with localized advanced NSCLC. This could reflect immune-suppression as a consequence of treatment although the authors did not determine whether the splenic volume decrease was related to patient outcome or correlate it to the efficacy of chemotherapy. In particular, they found that a significant decrease in spleen volume was observed between baseline and Week 2 for patients treated with concomitant carboplatin/paclitaxel and cisplatin/etoposide and a further decrease occurred by Week 4 compared with baseline in the carboplatin/paclitaxel treatment group, whereas in patients treated with cisplatin/etoposide, the splenic volume returned to above baseline levels.

In contrast, Jung et al. [14] found that splenomegaly was observed in 67% of patients after 12 cycles of oxaliplatin-based chemotherapy; moreover, a sudden increase in splenic size was evident in some after 6 cycles of chemotherapy. However, the clinical significance of such splenic volume change remains uncertain.

Susok et al. [16] decided to investigate the effect of immunotherapy on spleen volume in patients with advanced melanoma. When compared with the baseline, they observed after 3 months a significant increase of median spleen volume under treatment with immune checkpoint inhibitors due to enhanced accumulation and proliferation of immune cells. The increase of spleen size observed in this study was significantly associated only with the use of ipilimumab and its combination with nivolumab.

Only a few studies have investigated the response of tumour types other than melanoma to immunotherapy with 18F-fluorodeoxyglucose positron emission tomography–computed tomography (18F-FDG PET/CT), especially non-small cell lung cancer, and some are case reports [22–27]. In a recent study assessing response of NSCLC to nivolumab [23], 24 patients were investigated at baseline and 1 month after the start of treatment. Response was determined using either morphological (RECIST 1.1) or PERCIST criteria, along with SUVmax, metabolic tumour volume (MTV) and total lesion glycolysis (TLG). Metabolic responses by 18F–FDG uptake (especially TLG) was associated with therapeutic response and survival at 1 month after nivolumab administration. However, they included a very small sample size and evaluated only early PET-CT. Metabolic splenic volume have not been reported specifically in the context of NSCLC and immunotherapy.

In this study, we hypothesize that treatment with pembrolizumab is associated with lymphocyte response, which could be reflected in changes in the splenic size. Studies have shown that pre-treatment lymphopenia or high neutrophil to lymphocyte ratio [28] is associated with shorter time to disease progression, while the development of lymphopenia during treatment has been observed with increased drug-related toxicity [29]. By contrast, NSCLC patients that showed significant response to pembrolizumab treatment has been reported to show a significant increase in the percentage of lymphocytes in the peripheral blood [30]. We theorize that the latter could be associated with an increase in splenic volume.

The lack of any significant splenic volume change in our study may be related to a number of factors. The first lies in the mechanism of action of pembrolizumab. Pembrolizumab is a monoclonal antibody that binds to the PD-1 receptor that show high accumulation in organs such as the liver and spleen after administration [22, 31, 32]. It blocks the interaction with PD-L1 and PD-L2, releasing PD-1 pathway-mediated inhibition of the immune response, including the anti-tumor immune response, but may not have a significant effect on the splenic niche and structure. Second, peripheral lymphocytic expansion may not be associated with follicular expansion and splenic volume change. Indeed, in patients treated with platin-based chemotherapy, sinusoidal injury associated with such treatment has been associated with an increase in splenic size and is used as a secondary sign of liver damage [33, 34] rather than as a sign of treatment efficacy. Third, the splenic size is modulated by other factors such as physiological state and liver dysfunction, which may also occur in patients receiving pembrolizumab treatment.

There are limitations to this study. First, this study was retrospective. Nonetheless, this was a well-selected patient cohort with NSCLC and all received the same treatment. Second, the first CT follow-up available was at 12 weeks after commencing treatment and therefore we could not analyze any early changes in splenic volume.

## Conclusion

Our study did not reveal any significant temporal changes in the human spleen volume during immunotherapy and that the spleen volume cannot be used to differentiate responders from non-responders in patients receiving pembrolizumab for NSCLC. Further work is being undertaken to elucidate whether the CT radiomics/textures of the splenic volume may provide additional insights to pembrolizumab immunotherapy response.

## Supporting information

S1 Data (XLSX)
